# Expression of FLRT2 in Postnatal Central Nervous System Development and After Spinal Cord Injury

**DOI:** 10.3389/fnmol.2021.756264

**Published:** 2021-10-22

**Authors:** Juntan Li, Yo Shinoda, Shuhei Ogawa, Shunsuke Ikegaya, Shuo Li, Yukihiro Matsuyama, Kohji Sato, Satoru Yamagishi

**Affiliations:** ^1^Department of Organ and Tissue Anatomy, Hamamatsu University School of Medicine, Hamamatsu, Japan; ^2^Department of Environmental Health, School of Pharmacy, Tokyo University of Pharmacy and Life Sciences, Tokyo, Japan; ^3^Division of Integrated Research, Research Institute for Biomedical Sciences, Tokyo University of Science, Chiba, Japan; ^4^Department of Orthopedic Surgery, Hamamatsu University School of Medicine, Hamamatsu, Japan

**Keywords:** FLRT2, axon guidance, synaptic plasticity, development, spinal cord injury

## Abstract

Fibronectin and leucine-rich transmembrane (FLRT) proteins are necessary for various developmental processes and in pathological conditions. FLRT2 acts as a homophilic cell adhesion molecule, a heterophilic repulsive ligand of Unc5/Netrin receptors, and a synaptogenic molecule; the last feature is mediated by binding to latrophilins. Although the function of FLRT2 in regulating cortical migration at the late gestation stage has been analyzed, little is known about the expression pattern of FLRT2 during postnatal central nervous system (CNS) development. In this study, we used *Flrt2-LacZ* knock-in (KI) mice to analyze FLRT2 expression during CNS development. At the early postnatal stage, FLRT2 expression was largely restricted to several regions of the striatum and deep layers of the cerebral cortex. In adulthood, FLRT2 expression was more prominent in the cerebral cortex, hippocampus, piriform cortex (PIR), nucleus of the lateral olfactory tract (NLOT), and ventral medial nucleus (VM) of the thalamus, but lower in the striatum. Notably, in the hippocampus, FLRT2 expression was confined to the CA1 region and partly localized on pre- and postsynapses whereas only few expression was observed in CA3 and dentate gyrus (DG). Finally, we observed temporally limited FLRT2 upregulation in reactive astrocytes around lesion sites 7 days after thoracic spinal cord injury. These dynamic changes in FLRT2 expression may enable multiple FLRT2 functions, including cell adhesion, repulsion, and synapse formation in different regions during CNS development and after spinal cord injury.

## Introduction

The mammalian cerebral cortex is a highly organized laminar structure with six layers. During development, the cortical plate develops in an inside-out pattern, i.e., later-born neurons migrate outward through neurons born earlier. After arriving at their final destinations, neurons extend their axons and establish synaptic connections with neurons in other layers and regions, allowing each layer and region to exhibit characteristic features. Axon guidance molecules such as Netrin, Ephrins, Semaphorins, and Slits are involved in organizing the precise positioning and connection of neurons (Chen et al., 2008; Torii et al., 2009; Gonda et al., 2020; Yamagishi et al., 2021). However, the molecular mechanisms involved in axonal guidance and synaptogenesis during development and in response to nerve injury have not yet been fully elucidated.

Fibronectin and leucine-rich transmembrane (FLRT) protein family members are type I transmembrane proteins consisting of extracellular leucine-rich repeat sequences (LRRs), type III fibronectin (FNIII) domains, and cytoplasmic tails with Rnd1 binding motifs (Lacy et al., 1999; Ogata et al., 2007). FLRTs play important roles in cell–cell adhesion, cell migration, axon guidance, and vasculogenesis during development (Akita et al., 2016; Peregrina and Del Toro, 2020; Yamagishi et al., 2021), and they also play pivotal roles in interneuron development and final cortical allocation *in vivo* (Fleitas et al., 2021). Fibronectin and leucine-rich transmembranes can be induced by FGF-mediated signaling cascade in many tissues during mouse embryogenesis, such as in the midbrain/hindbrain boundary, the apical ectodermal ridge, and the developing sclerotome (Haines et al., 2006). Fibronectin and leucine-rich transmembranes also mediate intercellular adhesion through heterophilic interaction with latrophilins expressed on the surface of adjacent cells, as well as homophilic interactions (Muller et al., 2011; O’Sullivan et al., 2012; Seiradake et al., 2014; Jackson et al., 2015; Lu et al., 2015; Del Toro et al., 2020). In the process of cell migration and axon guidance, FLRTs interact with UNC5s to contribute to repulsion in neuron guidance (Yamagishi et al., 2011; Seiradake et al., 2014; Jackson et al., 2015). Moreover, FLRT2 and FLRT3 also play important roles in embryonic development. In mouse, *Flrt3* mutants are lethal due to dysfunction in several aspects of embryonic development: highly disorganized basement membrane in the anterior visceral endoderm region; defects in headfold fusion; and failure of the lateral edges of the ventral body wall to fuse, leading to cardia bifida (Egea et al., 2008; Maretto et al., 2008). Mouse embryos lacking *FLRT2* expression arrest at mid-gestation due to cardiac insufficiency (Muller et al., 2011). Conditional knockout mice lacking FLRT2 in vascular endothelial cells exhibit embryonic lethality at the mid-gestation stage with systemic congestion and hypoxia (Tai-Nagara et al., 2017). FLRT1 and FLRT3 promote increases in neurite number and length (Wheldon et al., 2010). FLRT2 regulates dendritic spine density in CA1 neurons (Schroeder et al., 2018). Crystal structures of FLRT proteins imply that distinct binding sites can trigger adhesive and/or repulsive signals in the same receiving cell, leading to an integrative response (Seiradake et al., 2014). Recent work showed that the TEN2–LPHN3–FLRT3 ternary complex is important for the formation of synaptic properties (Sando et al., 2019; Li et al., 2020).

In humans, FLRT2 is expressed in pancreas, skeletal muscle, brain, and heart (Lacy et al., 1999). During embryogenesis, FLRT2 is expressed in endothelial cells in the placental labyrinth (Tai-Nagara et al., 2017). During cortical development, FLRT2 expression is confined to the cortical plate, especially in deep layers, starting from E15.5 (Yamagishi et al., 2011). However, the expression pattern of FLRT2 in the brain in later postnatal stages remains largely unknown. In this study, using *Flrt2-LacZ* knock-in (KI) and Nestin-Cre;FLRT2^lx/lx^ cKO mice, we investigated the detailed expression pattern of FLRT2 in the brain of neonatal and adult mice. In addition, we also performed electrophysiological analyses of synaptic function by deleting FLRT2 in excitatory neurons in the hippocampus using Emx1-Cre mice. Finally, we analyzed FLRT2 expression in response to spinal cord injury.

## Materials and Methods

### Animals

*Flrt2-LacZ* KI mice were obtained and maintained on a CD1 background (Tai-Nagara et al., 2017). P0, P7, and adulthood (>6 weeks old) *Flrt2-LacZ* KI mice were used for LacZ staining. For electrophysiological and immunohistochemistry experiments, *Flrt2*^lox/lox^ mice (Tai-Nagara et al., 2017) were crossed with Emx1-Cre (Iwasato et al., 2000) and Nestin-Cre (Isaka et al., 1999), respectively. For validating the conditional allele, FLRT2^+/–^ (Yamagishi et al., 2011), FLRT2^lox/+^ and Nestin-Cre mice were crossed. Male mice aged 2–4 months were used for electrophysiological experiments. Primers pairs for genotyping were as follows: *Flrt2-LacZ* KI mice, 5′-TTACACAG ACTGCCACATCC, 5′-CCTGCAGCCCAAGCTGATCC and 5′-GAGCCCACCTGACATTATCC; (2) *Flrt2*^lox/lox^ mice, 5′-GT GGAAGGAAGGAATTGTCTCAGG and 5′-GGAGCCAGGTT GGCAGGAGTTGGC; and (3) Cre mice, 5′-GCCTGCAT TACCGGTCGATGCAACGA and 5′-GTGGCAGATGGCG CGGCAACACCATT-3′. All animal experiments were approved by the Animal Research Committee of Hamamatsu University School of Medicine and were carried out in accordance with the in-house guidelines for the care and use of laboratory animals of the university.

### *LacZ* Staining

*LacZ* staining was performed as previously reported (Tai-Nagara et al., 2017). Briefly, mice were anesthetized with a mixture containing medetomidine hydrochloride, midazolam, and butorphanol tartrate (0.1 ml/10 g delivered by intraperitoneal injection), and then fixed via cardiac perfusion with 0.01 M sodium phosphate-buffered saline (pH 7.4) for 1 min followed by 4% paraformaldehyde in 0.01 M PBS (pH 7.4) for 5 min. The brains were dissected and placed in cold PBS. Then, the brains were embedded in BSA (Sigma-Aldrich, MO, United States) solution cross-linked with glutaraldehyde (Nacalai Tesque Inc, Kyoto, Japan). After solidification, sagittal and coronal sections were cut at a thickness of 100 μm on a Vibratome (Dosaka EM Co Ltd, Kyoto, Japan), and then stained at 37°C overnight for X-gal (5-bromo-4-chloro-3-indolyl-β-galactoside) (Fuji-Wako, Osaka, Japan). Images of sections were acquired using a transmission light microscope (Eclipse E600; Nikon, Tokyo, Japan).

### Immunostaining

Immunostaining of tissue sections was performed as described previously (Ikegaya et al., 2020) with some modifications. Briefly, the mice were deeply anesthetized and intracardially perfused with phosphate-buffered saline (PBS), and then the brain and spinal cord were dissected. Dissected tissues were immediately frozen with dry ice. Sagittal and coronal sections (20 μm thickness) were prepared using a cryostat and stored at −80°C until use. Before staining, the sections were fixed in 2% PFA/PBS for 5 min, washed with PBS, and permeabilized in 0.3% Triton X-100/PBS for 5 min. The permeabilized sections were incubated for 1 h at room temperature with blocking solution containing either 3%BSA or 10% donkey serum in 0.1% Triton X-100/PBS, followed by incubation with primary antibodies in either 3% BSA or 10% donkey serum/0.1% Triton X-100/PBS overnight at 4°C. The sections were then incubated with Alexa Fluor-conjugated secondary antibodies for 30 min at room temperature, followed by nuclear staining with DAPI. Images of the sections were acquired with a confocal microscope (TCS SP8; Leica, Wetzlar, Germany). The primary antibodies used were goat anti-FLRT2 (1:350; R&D Systems, Minneapolis, MN, United States), rabbit anti-PSD95 (1:200; Cell Signaling Technology, Danvers, MA, United States), guinea pig anti-VGLUT1 (1:1000; Millipore; Darmstadt, Germany) and mouse anti-GFAP-Cy3 conjugated (1:500; Sigma-Aldrich, Burlington, MA, United States) antibodies. Secondary antibodies were donkey anti-rabbit, donkey anti-goat and goat anti-guinea pig (conjugated to Alexa Fluor 488 and 568, respectively; Thermo Fisher Scientific, Waltham, MA, United States), used at a dilution of 1:500.

### Electrophysiology

Electrophysiology was performed as reported previously with minor modifications (Ishii et al., 2021). Briefly, deeply anesthetized mice were decapitated and their brains were rapidly removed into ice-cold high-sucrose Ringer’s solution (234 mM sucrose, 2.5 mM KCl, 1.25 mM NaH_2_PO_4_, 10 mM MgSO_4_, 0.5 mM CaCl_2_, 26 mM NaHCO_3_, and 11 mM glucose; pH 7.5). Hippocampi were transversely sliced (thickness, 400 μm) on a vibratome Pro-7 (Dosaka, Kyoto, Japan), and slices were recovered in ACSF (125 mM NaCl, 2.5 mM KCl, 1.25 mM NaH_2_PO_4_, 1 mM MgCl_2_, 2 mM CaCl_2_, 26 mM NaHCO_3_, and 11 mM D-glucose; pH 7.5) at room temperature for at least 2 h. All solutions were constantly bubbled with 95% O_2_/5% CO_2_. Recovered slices were placed in a recording chamber and immersed in ACSF at 26°C. A bipolar platinum–iridium stimulation electrode (WPI, Sarasota, FL, United States) was placed in the CA1 stratum radiatum region. Field excitatory postsynaptic potentials (fEPSPs) were recorded from the CA1 stratum radiatum using an Ag/AgCl recording electrode placed in a glass pipette (Harvard Apparatus, Holliston, MA, United States) filled with ACSF following a 0.05 Hz test pulse generated by pulse generator Master-8 (AMPI, Jerusalem, Israel). Long-term potentiation (LTP) was induced by using theta-burst stimulation (TBS) consisting of four trains with 10 s intervals between trains; each train had five bursts separated by 200 ms, and each burst included four pulses delivered at 100 Hz at 20% of maximal stimulus intensity. Data were amplified using a MultiClamp 700A (Molecular Devices, San Jose, CA, United States), and digitized at 10 kHz and filtered at 2 kHz using a Digidata 1440 system with the pCLAMP10 software (Molecular Devices).

### Spinal Cord Injury

Eight- to nine-week-old *Flrt2-LacZ* KI and Nestin-Cre;FLRT2^lx/lx^ cKO mice were anesthetized with medetomidine hydrochloride, midazolam, and butorphanol tartrate (0.1 ml/10 g). Spinal cord injury at the tenth thoracic vertebra (T10) was induced using an Infinite Horizons impactor (IH impactor; Muromachi, Tokyo, Japan) with an impact force of 60 kdyn, as described previously (Arima et al., 2014). Animals in the sham group were conducted to laminectomy alone. These animals were sacrificed at 3, 7, 14, and 28 days post-injury.

### Western Blot Analysis

Brain lysates were obtained as previously described (Yamagishi et al., 2011). Glycoproteins were pulled down from 1000 μg of protein lysate using wheat germ agglutinin (lectin) agarose beads (Sigma). Western blots in lectin pull downs or total cell lysates were performed as indicated: goat anti-FLRT2 antibody (1:1000; R&D Systems, Minneapolis, MN, United States) and rabbit anti-tubulin antibody (1:1000; Thermo Fisher Scientific, Waltham, MA, United States).

## Results

### General Expression Patterns of FLRT2 in the Brain at P0, P7, and Adulthood

**FIGURE 1:**
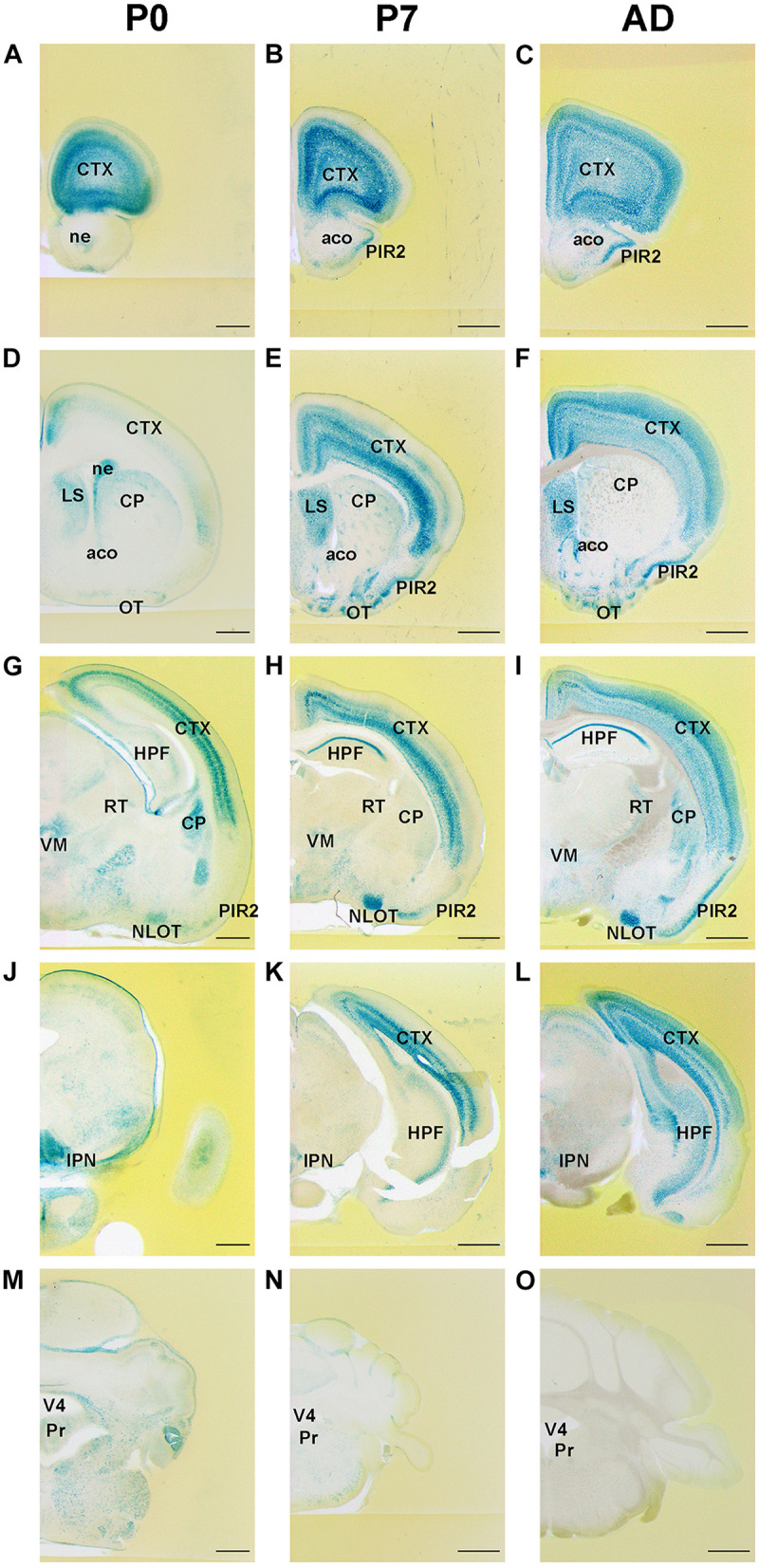
Expression pattern of *Flrt2-lacZ* in the postnatal stages of brain development. *Flrt2-lacZ* expression was observed on the planes of the prefrontal cortex **(A–C)**, LS **(D–F)**, HPF **(G–I)**, midbrain **(J–L)**, and cerebellum **(M–O)** at P0 **(A,D,G,J,M)**, P7 **(B,E,H,K,N)**, and adulthood **(C,F,I,L,O)**. aco, anterior commissure, olfactory limb; ACB, nucleus accumbens; CP, caudate putamen; CTX, cerebral cortex; HPF, hippocampal formation; IPN, interpeduncular nucleus; LS, lateral septal nucleus; ne, neuroepithelium; NLOT, nucleus of the lateral olfactory tract; OT, olfactory tubercle; PIR2, piriform area, pyramidal layer; Pr, prepositus nucleus; RT, reticular nucleus of the thalamus; VM, ventral medial nucleus of thalamus; V4, fourth ventricle. Scale bars, 500 μm **(A,D,G,J,M)**; 1 mm **(B,C,E,F,H,I,K,L,N,O)**. *n* = 3, 3, and 4 for P0, P7 and adulthood, respectively.

Expression patterns of FLRT2 in the developing brain were examined using *Flrt2-LacZ* KI heterozygote mice at P0, P7, and adulthood (>6 weeks old). Although LacZ staining was very intense in homozygotes, we used heterozygotes for the analysis because the homozygotes were embryonic lethal (Muller et al., 2011; Tai-Nagara et al., 2017). We observed intense FLRT2 expression in the anterior part of the cortex at P0 and P7 (Figures 1A,B). The expression decreased in adult mice (Figure 1C and Supplementary Figure 1). FLRT2 was expressed in the neural epithelium at P0, but not at P7 or adulthood (Figures 1D–F). By contrast, LacZ staining was observed in the piriform area (PIR), mainly in the pyramidal layer (PIR2) at P7 and adulthood, but not at P0. In the cortex, the deep layer neurons expressed FLRT2 at the early stages, but FLRT2 expression was expanded in the adult mice (Figures 1G–I and Supplementary Figure 2). In CA1 of the hippocampus, FLRT2 expression was prominent after P7. In the brain stem and cerebellum, except for several nuclei, the intensity of LacZ staining was much weaker than in the cortex (Figures 1J–O). We could observe weak staining in the Purkinje layer of the posterior cerebellum in the adult mice (Supplementary Figures 3A–C). In the amygdala, weak and moderate FLRT2 expression was observed in the lateral amygdala nucleus (LA) at P7 and adulthood, respectively. In addition, a shift from moderate to weak FLRT2 expression was observed in the basolateral amygdala nucleus, anterior part (BLAa) at P0, P7, and adulthood (Supplementary Figures 3D–F). During development, intermediate staining was consistently observed in some areas of the pons, such as the parabrachial nucleus ventral lateral part (PBlv), parabrachial nucleus external lateral part (PBle), and locus ceruleus (LC) (Table 1).

**TABLE 1:** Distribution and intensity of FLRT2-LacZ expression in the mouse CNS.

	*Flrt2-LacZ* expression		
	
	P0	P7	adulthood
Telencephalon			
Olfactory system			
Olfactory bulb (OB)	−	−	−
Piriform area (PIR) layer 2	−	+ +	+ + +
Anterior olfactory nucleus (AON)	−	−	+
Olfactory tubercle (OT)	+	+ +	+ +
Nucleus of the lateral olfactory tract (NLOT)	+ +	+ + +	+ + +
Taenia tecta (TT) layer 2	+ +	+	+
Cerebral cortex			
Primary somatosensory area			
Layer I	−	−	−
Layer II/III	−	−	+
Layer IV	+ +	+	+ + +
Layer V	+ +	+ +	+ +
Layer VI	+	+ + +	+ +
Agranular insular area	+ +	+ +	+
Basal forebrain			
Bed nucleus of stria terminalis (BST)	−	+	+
Endopiriform nucleus (EP)	−	+	+
Claustrum (CLA)	−	+	+
Substantia innominata (SI)	−	+	+
Septum			
Lateral septal nucleus (LS)	+ +	+ +	+ +
Medial septal nucleus (MS)	−	−	−
Basal ganglia			
Caudate putamen (CP)	+ + +	+ +	+
Nucleus accumbens (ACB)	+ + +	+	+
Globus pallidus external segment (GPe)	+	−	−
Globus pallidus internal segment (GPi)	+	−	−
Subthalamic nucleus (STN)	+	+	+
Substantia nigra reticular part (SNr)	−	+	−
Substantia nigra compact part (SNc)	−	+	+
Hippocampal formation (HPF)			
CA1			
oriens	−	+	+
pyramidal layer	+	+ + +	+ + +
radiatum	−	−	−
lacunosum-moleculare	−	−	−
CA2			
oriens	−	−	+
pyramidal layer	−	+ + +	+ + +
radiatum	−	+	+
lacunosum-moleculare	−	−	+
CA3			
oriens	−	−	+
pyramidal layer	−	+	+
radiatum	−	−	+
lacunosum-moleculare	−	−	+
Dentate gyrus (DG)			
polymorph layer	+	+	+
granule cell layer	−	−	+
molecular layer	−	−	+
Amygdala			
Lateral amygdala nucleus (LA)	−	+	+ +
Basolateral amygdala nucleus, anterior part (BLAa)	+ +	+ +	+
Basolateral amygdala nucleus, posterior part (BLAp)	−	+ +	+
Basolateral amygdala nucleus, ventral part (BLAv)	−	+ +	+
Cortical amygdala area (COA)	+ +	+ +	+ +
Piriform-amygdala area (PAA)	−	+	+
Diencephalon			
Medial habenula (MH)	+ +	+	+
Lateral habenula (LH)	+ +	+	+
Reticular nucleus of the thalamus (RT)	−	−	+
Ventral medial nucleus of the thalamus (VM)	+ + +	+	+
Hypothalamus			
Paraventricular hypothalamic nucleus (PVH)	−	−	+
Ventromedial hypothalamic nucleus (VMH)	+ +	+ +	+
Arcuate hypothalamic nucleus (ARH)	−	−	+
Lateral hypothalamic area (LHA)	+	+	+
Suprachiasmatic nucleus (SCH)	−	−	+
Midbrain			
Superior colliculus superficial gray layer (SCsg)	−	+	+ +
Superior colliculus intermediate gray layer (SCig)	−	+	+
Superior colliculus deep gray layers (SCdg)	−	−	+
Interpeduncular nucleus (IPN)	+ + +	+ +	+
Periaqueductal gray (PAG)	+	+	+
Midbrain reticular nucleus (MRN)	+ +	+	+
Hindbrain			
Cerebellum granular layer	−	−	−
Cerebellum Purkinje layer	−	−	+
Cerebellum molecular layer	−	−	−
Pontine reticular nucleus (PRNr)	+	+	−
Parabrachial nucleus ventral lateral part (PBlv)	+ +	+ +	+ +
Parabrachial nucleus external lateral part (PBle)	+ +	+ +	+ +
Locus ceruleus (LC)	+ +	+	+
Laterodorsal tegmental nucleus (LDT)	+	+	+
Dorsal tegmental nucleus (DTN)	+ + +	+ + +	+ +

Within the basal forebrain, no LacZ staining was observed at P0 and a weak expression was observed at both P7 and adulthood (Table 1). In the diencephalon, LacZ staining was observed in the medial habenula (MH), the lateral habenula (LH) (Supplementary Figures 4A–C), and the ventromedial hypothalamic nucleus (VMH) (Table 1). In addition, LacZ staining was observed in the interpeduncular nucleus (IPN) at P0, P7, and adulthood (Supplementary Figures 4D–F) and the midbrain reticular nucleus (MRN) (Table 1).

### Postnatal FLRT2 Expression in the Primary Somatosensory Area of Cortex and During Hippocampal Formation

**FIGURE 2:**
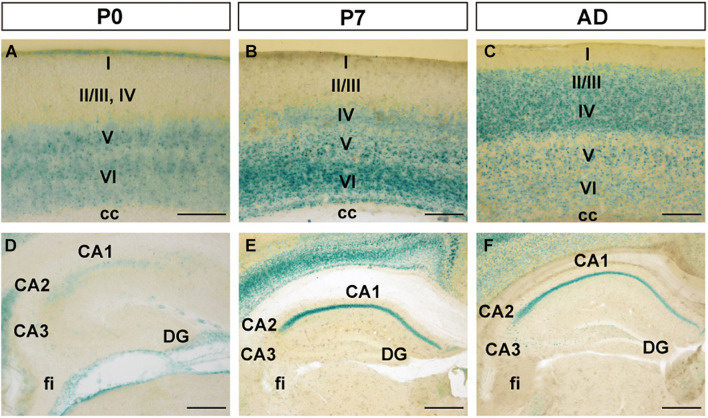
FLRT2 expression in the cortical region (CTX) and during hippocampal formation (HPF) at P0, P7, and adulthood. **(A–C)** In the cerebral cortex, FLRT2 was expressed at deep layers at P0 **(A)**, followed by layer IV at P7 **(B)** and superficial layers II/III at adulthood **(C)**. **(D–F)** In the hippocampus, FLRT2 was expressed at a lower level in the CA1 at P0 **(D)**, becoming more intense at P7 **(E)** and adulthood **(F)**, concentrated in the pyramidal layer of CA1 but not CA2, CA3, or DG. cc, corpus callosum; CA1, Ammon’s horn field 1; CA2, Ammon’s horn field 2; CA3, Ammon’s horn field 3; DG, dentate gyrus; I–VI, layers of cortical region. Scale bars, 100 μm **(A)**; 200 μm **(B,C,D)**; 500 μm **(E,F)**. *n* = 3, 3, and 4 for P0, P7 and adulthood, respectively.

As age increased, FLRT2 expression in the primary somatosensory area of cerebral cortex gradually spread from the deep layer to the superficial layers, except for layer I (Figures 2A–C). In layer II/III, moderate FLRT2 expression was observed at adulthood but not at P0 or P7, consistent with our previous report (Yamagishi et al., 2011). In adulthood, the strongest expression was observed in layer IV, where thalamocortical axons project. In layer V, moderate expression was observed at adulthood, whereas expression in this region was stronger at P0 and P7. In layer VI, as age increased, expression decreased from strong to moderate, as in layer V (Figures 2A–C). In addition, we also observed intense FLRT2 expression in the rostral part of the cerebral cortex at P0, but not at P7 or adulthood (Supplementary Figure 1). FLRT2 was expressed in Ctip2 + layer V at P0 (Supplementary Figure 2). In addition, we observed strong expression of FLRT2 on the pia surface at P0 that decreased at P7 and adulthood (Figures 2A–C).

During formation of CA1 of the hippocampus, although weak expression of FLRT2 was observed at P0, strong and specific expression was observed in pyramidal cell layers at P7 and adulthood (Figures 2D–F). In CA2, weak expression was observed at P0, and moderate expression was observed in the pyramidal cell layer at P7 and adulthood. In CA3 and DG, very faint expression was observed at all three stages.

### Postnatal FLRT2 Expression in the Caudate Putamen, Lateral Septal Nucleus, and Nucleus of the Lateral Olfactory Tract

**FIGURE 3:**
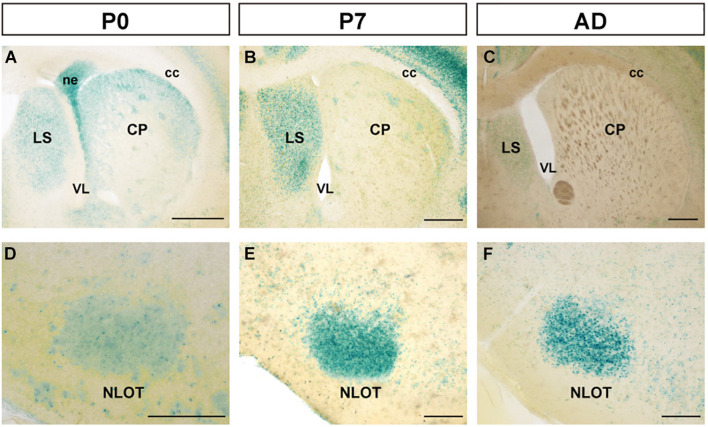
Expression pattern in the caudate putamen (CP), lateral septal nucleus (LS), and nucleus of the lateral olfactory tract (NLOT) at P0, P7, and adult mouse. **(A–C)** In the caudate putamen (CP), FLRT2 signals decreased during development. Note that strong expression was observed in the neuroepithelium at P0 **(A)** and the lateral septum at P7 **(B)**. **(D–F)** In the nucleus of the lateral olfactory tract (NLOT), intermediate expression of FLRT2 was observed at P0 **(D)**, and high expression was observed at P7 **(E)** and adulthood **(F)**. cc, corpus callosum; CP, caudate putamen; LS, lateral septal nucleus; ne, neuroepithelium; NLOT, nucleus of the lateral olfactory tract; VL, lateral ventricle. Scale bars, 500 μm **(A–C)**; 200 μm **(D–F)**. *n* = 3, 3, and 4 for P0, P7 and adulthood, respectively.

At P0, FLRT2 was widely expressed in the caudate putamen (CP) and in sparse clusters of the CP (Figure 3A). At P7, the clusters of medium LacZ staining were more prominent (Figure 3B). However, expression was dramatically reduced at adulthood (Figure 3C). On the other hand, in the lateral septal nucleus (LS), moderate expression was also observed at P0, P7, and adulthood (Figures 3A–C). Strong expression was observed in the neuroepithelium (ne) on the lateral side of the lateral ventricle at P0, but not at P7 or adulthood (Figures 3A–C). In addition, intense LacZ expression was observed in nucleus of the lateral olfactory tract (NLOT) at P7 and adulthood (Figures 3D–F).

### FLRT2 Expression in the Ventral Medial Nucleus, the Reticular Nucleus of the Thalamus, and the Dorsal Tegmental Nucleus

**FIGURE 4:**
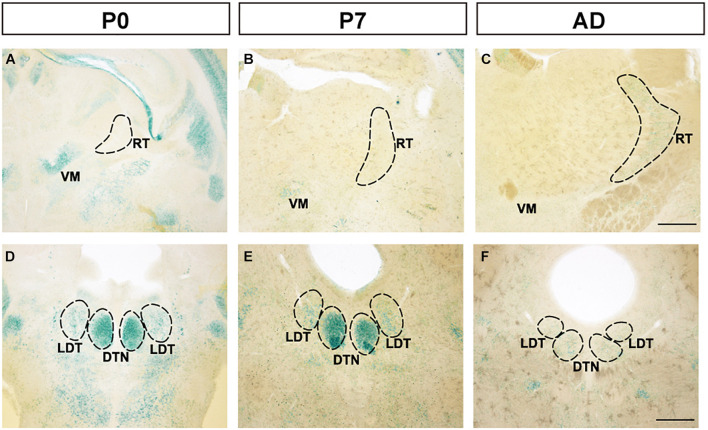
Expression patterns in the thalamus and midbrain at P0, P7, and adulthood, revealed by X-gal staining. **(A–C)** FLRT2 expression levels changed from absent to weak in the ventral medial nucleus of the thalamus (VM) and from strong to weak in the reticular nucleus of the thalamus (RT) during development. **(D–F)** In the dorsal tegmental nucleus (DTN), expression of FLRT2 was high at P0 and P7, but decreased by adulthood. DTN, dorsal tegmental nucleus; LDT, laterodorsal tegmental nucleus; RT, reticular nucleus of the thalamus; VM, ventral medial nucleus of the thalamus. Scale bars, 500 μm **(A–F)**. *n* = 3, 3, and 4 for P0, P7 and adulthood, respectively.

Although strong expression was observed at the ventral medial nucleus of the thalamus (VM) at P0, expression decreased at P7 and adulthood (Figures 4A–C). Additionally, in the reticular nucleus of the thalamus (RT), no expression was observed at P0 or P7, but weak expression was observed at adulthood. In the dorsal tegmental nucleus (DTN) in the midbrain, strong expression was observed at P0 and P7, but at adulthood, expression was weakened (Figures 4D–F). Expression in the laterodorsal tegmental nucleus (LDT) was constantly weaker than in DTN. In other areas of the thalamus and midbrain, FLRT2 expression level was very low (Figures 1G–L and Table 1).

### FLRT2 Is Expressed in Pre- and Postsynapses in the CA1, but Is Dispensable for CA3-CA1 Schaffer Collateral Synaptic Transmission

**FIGURE 5:**
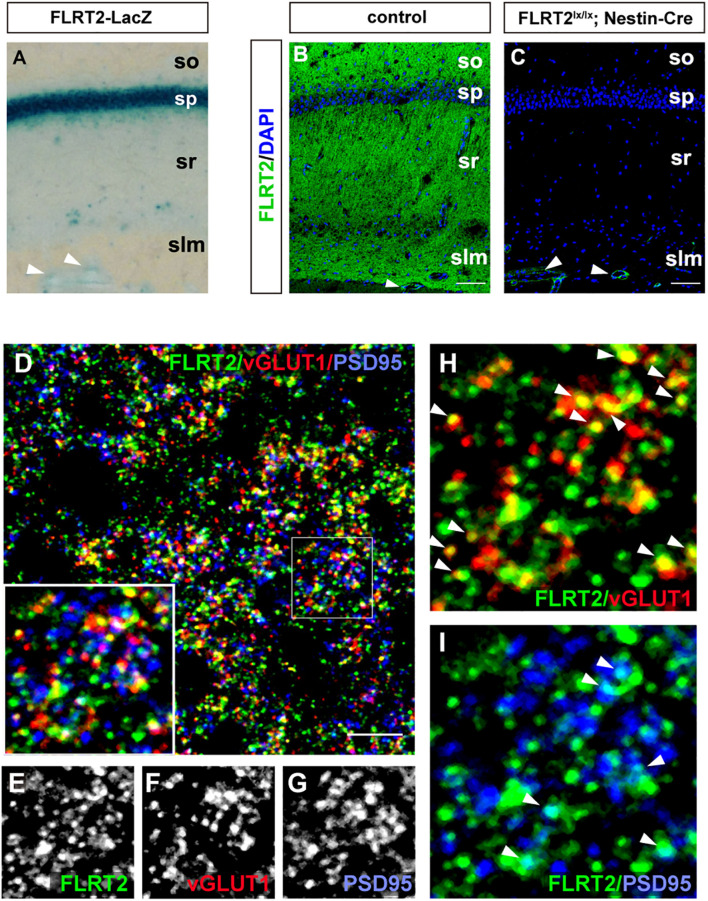
Synaptic localization of FLRT2 in the CA1 of adult mice. **(A)** FLRT2-*LacZ* expression was strong in the pyramidal layer. **(B)** FLRT2 protein expression was distributed in the layers of so, sr, and slm but not in sp. **(C)** FLRT2 was specifically knocked out in CA1 neurons in conditional knockout mice, but not in blood vessels (arrowheads). **(D–I)** FLRT2 partly colocalized with vGLUT1 and PSD95 (arrowheads in **H** and **I**). Inset: higher magnification. so, stratum oriens; sp, stratum pyramidale; sr, stratum radiatum; slm, stratum lacunosum-moleculare. Scale bars, 100 μm **(B,C)**, 5 μm **(D)**. *n* = 4 for *Flrt2-LacZ* KI adulthood mice.

Because LacZ staining was confined to the CA1 region in the adult hippocampus (Figures 2F, 5A), we next sought to visualize FLRT2 protein expression by immunostaining. FLRT2 immunoreactivity was high in the layers of the stratum oriens (SO), stratum radiatum (SR), and stratum lacunosum-moleculare (SLM), but not in the stratum pyramidale (SP) (Figure 5B). Reactivity was dramatically diminished in the Nestin-Cre;FLRT2^lx/lx^ cKO as expected, indicating the specificity of the antibody (Figure 5C). Higher magnification revealed that in the SR, FLRT2 localized in globular dot patterns and was partially colocalized with the presynapses (vGLUT1; 30.6%) and postsynapses (PSD95; 15.7%), respectively (Figures 5E–I). The postsynaptic localization was consistent with previous findings (Schroeder et al., 2018). In addition, FLRT2 was expressed in the vascular endothelial cells on the border between the SLM and DG, and this expression could not be abolished by crossing with Nestin-Cre mice. We could not detect any signal in the SR or SO of the cKO mice, indicating that the endothelial cells of capillary vessels do not express FLRT2. Recombination of FLRT2 allele by Nestin-Cre was also confirmed by western blotting (Supplementary Figure 5).

Next, we investigated electrophysiological properties to determine whether FLRT2 expression on postsynapses is involved in synaptic transmission in the CA3–CA1 Schaffer collateral pathway (Supplementary Figures 6A–D). However, relative to the control group, we could not detect any alteration in either the amplitude or slope of fEPSP in the Emx1-Cre; FLRT2^lx/lx^ cKO mice (Supplementary Figures 6A,B). Furthermore, we observed no significant differences in paired-pulse facilitation before or after LTP induction or in TBS-induced LTP (Supplementary Figures 6C,D). These results indicate that FLRT2 is dispensable for CA3-CA1 Schaffer collateral synaptic transmission.

### FLRT2 Expression Is Elevated in Response to Spinal Cord Injury

**FIGURE 6:**
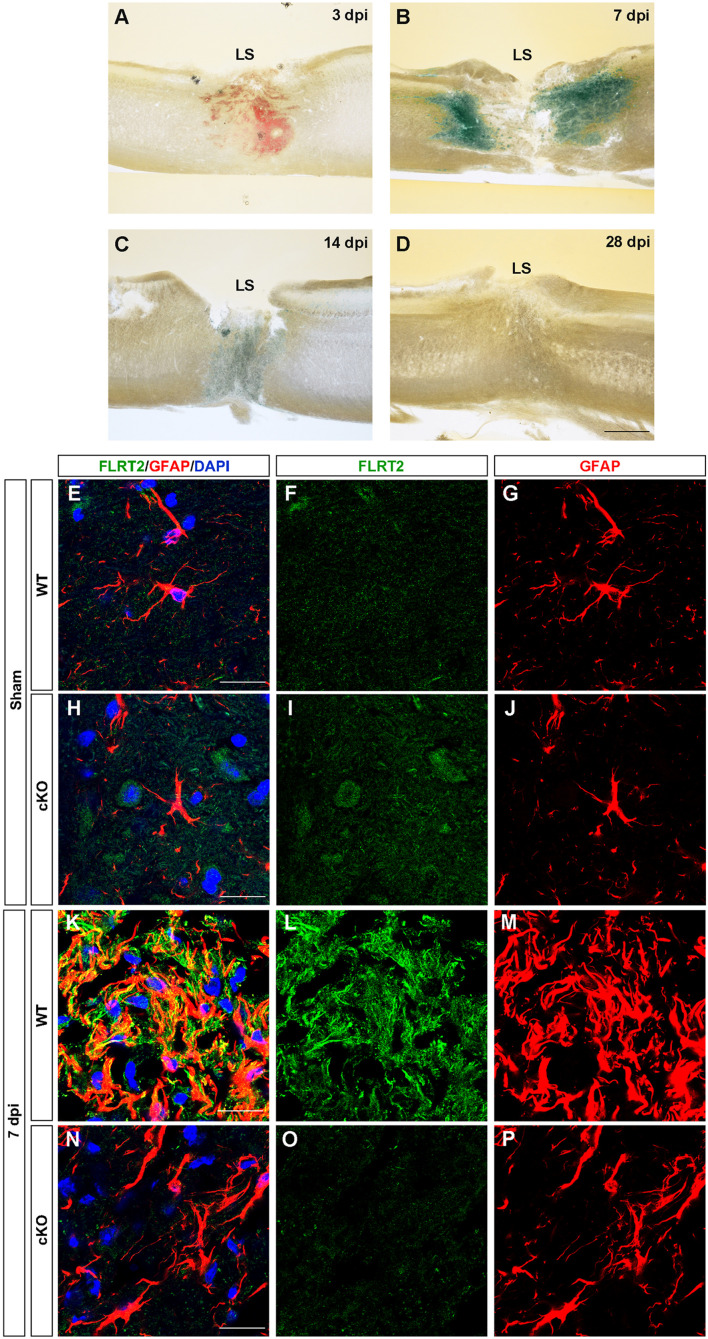
FLRT2 is strongly expressed in astrocytes 7 days after spinal cord injury. **(A–D)** Sagittal slices 3, 7, 14, and 28 days after spinal cord injury, subjected to X-gal staining. Strong FLRT2 expression was observed at 7 dpi and decreased rapidly. **(E–P)** FLRT2 was expressed in GFAP + astrocytes at 7 dpi in wild type mice. GFAP induction after spinal cord injury was decreased in FLRT2 cKO. *n* = 3 for each group. dpi, days post-injury; LS, lesion site. Scale bars, 500 μm **(A–D)**; 20 μm **(E–M)**.

In the spinal cord, we observed down-regulation of FLRT2 during development (Supplementary Figures 3G–I). Next, because many axon guidance molecules, including FLRT3 and RGMa, are upregulated upon central and peripheral nervous system injury (Robinson et al., 2004; Tsuji et al., 2004; Hata et al., 2006; Yamada et al., 2019), we investigated whether FLRT2 is upregulated after spinal cord injury. FLRT2 was highly upregulated around lesion sites 7 days after thoracic spinal cord injury (Figures 6A–D); weak expression was maintained until day 14, but was no longer visible by day 28 (Figures 6C,D). In the uninjured spinal cord gray matter, FLRT2 expression was weak in wild type mice (Figures 6F,I), which is consistent with the result of LacZ staining (Supplementary Figure 3I), and a relatively small number of GFAP + cells was observed (Figures 6G,J). Expression of FLRT2, induced at the subacute phase, was localized on GFAP + reactive astrocytes (Figures 6K–M). Interestingly, the provocation of GFAP at 7 days after the injury was decreased in Nestin-Cre; FLRT2^lx/lx^ cKO mice (Figures 6N–P). These results suggest that FLRT2 serves as a repulsive guidance molecule like RGMa, inhibits CNS regeneration, acts as an adhesive molecule, and contributes to form glial scar.

## Discussion

In this study, we observed FLRT2 expression in multiple regions of the CNS at these three time points (Figure 1). Confined FLRT2 expression in CA1 at the postnatal stage suggested a pivotal role for FLRT2 in synaptic function (Figure 2). In addition, FLRT2 expression was confined to the CA1 region and partly localized on pre- and postsynapses whereas only few expression was observed in CA3 and dentate gyrus (DG) (Figure 5). Furthermore, astrocytes transiently expressed FLRT2 in the penumbra region after spinal cord injury (Figure 6).

In the early postpartum period, the cortex is still rapidly developing. We observed that FLRT2 expression was confined to deep layers of the cortex at P0, consistent with our previous report (Yamagishi et al., 2011). Because FLRT2 acts as both a repulsive guidance cue and an adhesive molecule (Yamagishi et al., 2011), it may play a role in fine tuning of cortical circuits. Eventually, in adult mouse brain, cells expressing FLRT2 appeared mainly in layer IV, which is occupied by spiny stellate cells (Lefort et al., 2009). This region is where thalamocortical axons predominantly terminate (White and Keller, 1989), suggesting that FLRT2 may play an important role in the formation of neural networks. Interestingly, we observed expression of FLRT2 in the RT only in the brains of adult mice. Because it mainly receives projections from the cortex, this may be intrinsically related to expression of FLRT2 in the cortex. On the other hand, in the VM, which receives afferent input from the basal ganglia and relays to the cerebral motor cortex (Kuramoto et al., 2015), the expression of FLRT2 at P7 and adulthood was much lower than at P0. In addition, we also observed reduced FLRT2 expression during development in the CP, a part of the basal ganglia. Based on these observations, FLRT2 may play a key role not only in the development of specific brain regions but also in intricate inter-regional connections such as the cortico-basal ganglia-thalamo-cortical loop.

Notably, we observed FLRT2 expression in vessels in the hippocampus that could not be abolished by Nestin-Cre (Figure 5C). Our previous studies showed that mice lacking FLRT2 in vascular endothelial cells exhibit embryonic lethality at mid-gestation with systemic congestion and hypoxia (Tai-Nagara et al., 2017). Notably, FLRT3 is a newly identified member of a family of axon guidance-related factors that participate in VEGF-signaling and regulation of endothelial cell functions (Jauhiainen et al., 2019). We observed that FLRT2 is expressed in the vascular endothelial cells of the hippocampus in adult mice, indicating that FLRT2 may play a key role in maintenance of vascular morphology and function at various stages of development. However, the function of FLRT2 in vascular endothelium in the CNS remains unclear and requires further study.

In contrast to weak expression in the hippocampal CA1 at P0, high expression in the pyramidal layer limited to CA1 began to be observed at P7 and was maintained till adulthood. Our results showed that FLRT2 is expressed by CA1 pyramidal neurons, consistent with the Allen Brain Atlas. Electrophysiology revealed that the CA1 postsynaptic localization of FLRT2 is dispensable in the Schaffer collateral synaptic connection (Supplementary Figure 6). Electrophysiological studies revealed that basal synaptic transmission of CA3-CA1 Schaffer collateral synapses in hippocampus did not altered in Emx1-Cre; FLRT2^lox/lox^ cKO, indicating dispensability of FLRT2. Consistent to our result, Cicvaric et al. reported that in haplodeficient *FLRT2*^+/–^ male mice don’t show any defect in synaptic transmission and plasticity in the hippocampal CA1, although *FLRT2*^+/–^ female mice showed reduced synaptic transmission and enhanced LTP because of decreased expression levels of EAAT2 and estrogen receptor β (Cicvaric et al., 2018). In the present study, we did not analyze female mice so that synaptic properties in the gender difference in the mutant mice are still unclear. On the other hand, knocking down postsynaptic FLRT2 in the CA1 caused decreased synaptic density at SC inputs, decreasing the frequency of EPSPs and the amplitude of AMPA and NMDA EPSCs (Schroeder et al., 2018). Because we used FLRT2 conditional knockout mice, there may have been developmental compensation by another molecule, e.g., FLRT1. Recent studies revealed that the TEN2–LPHN3–FLRT3 ternary complex plays a key role in the formation of synaptic properties (Sando et al., 2019; Li et al., 2020), but it remains unclear how FLRT2 plays a role in the complex. Further studies are required for clarify detailed function of FLRT2 in the synaptic transmission.

In terms of the olfactory system, FLRT2 expression changed from postpartum to adulthood in layer 2 of the PIR and NLOT. Layer 2 of the PIR is mainly composed of semilunar cells and superficial pyramidal cells. These two main types of primary neurons in the densely populated input layer (layer II) of the PIR have markedly different synaptic and firing characteristics, and they may provide the basis of coding strategies used to represent odors (Suzuki and Bekkers, 2006). This finding indicates that FLRT2 may participate in the coding strategy of PIR during the development of the nervous system. Rat studies have demonstrated that normal functioning of the olfactory system requires NLOT integrity (Vaz et al., 2017). Therefore, FLRT2 may be involved in the development of the olfactory system.

Expression of FLRT2 in the DTN in the early postpartum period was strong, but the expression dropped to a lower level in adulthood. The only known function of the DTN is to generate head-direction cell signals (Sharp et al., 2001; Bassett et al., 2007). In addition, a recent study showed that bilateral lesions of the DTN promote awakening at the expense of sleep (Chazalon et al., 2018). These findings indicate that FLRT2 contributes to establishment of the DTN neural network, and is involved in the sleep and awakening mechanism. Recent studies in rats indicate that the habenula is linked with LDT, either via direct reciprocal projections from/to the medial division of the lateral habenula (LHbM) or indirectly via the medial habenula (MHb)–interpeduncular nucleus (IPN) axis, supporting a functional role for LDT in the regulation of aversive behaviors, and for LHb as a master controller of ascending brainstem state-setting modulatory projection systems (Bueno et al., 2019). We also observed changes in FLRT2 expression in habenula and IPN, as observed in the LDT. Therefore, FLRT2 may play a role in formation of the neural network among the LDT, habenula, and IPN.

Here, we observed that FLRT2 is strongly expressed on GFAP + astrocytes, and the induction of GFAP was decreased in FLRT2 cKO mice at 7 days after spinal cord injury. The glial component of the scar after spinal cord injury consists of reactive astrocytes, NG2 + oligodendrocyte precursors, and microglia in the penumbra (Tran et al., 2018). Reactive astrocytes rapidly proliferate and densely populate the area around the lesion core within 7–10 days after glial scar formation (Wanner et al., 2013). In addition, a neutralizing antibody against repulsive guidance molecule a (RGMa) can dramatically facilitate locomotor improvement and axon regeneration after spinal cord injury in rats (Hata et al., 2006). Our results suggested that FLRT2 expressed on reactive astrocytes contributes to formation of glial scars as an adhesive molecule and induction of GFAP and inhibits axonal regeneration as a repulsive molecule. Thus, inhibiting FLRT2 function by applying neutralization antibodies may ameliorate scar formation after spinal cord injury and promotes CNS regeneration.

## Conclusion

FLRT2 exhibits different expression patterns at various stages from the early postnatal stage to maturity, indicating that it plays important roles in mouse development. In addition, FLRT2 expression is confined to the CA1 region and partly localized on pre- and postsynapses. We also observed FLRT2 signal at the boundary of lesion sites 7 and 14 days after spinal cord injury. These results provide some useful information about the role of FLRT2 in the developmental mechanisms of the CNS, the mechanism of regulation of synaptic plasticity, and other central neuropathy processes.

## Data Availability Statement

The original contributions presented in the study are included in the article/Supplementary Material, further inquiries can be directed to the corresponding author.

## Ethics Statement

The animal study was reviewed and approved by The Animal Research Committee of Hamamatsu University School of Medicine.

## Author Contributions

JL and SY designed, analyzed, and wrote the manuscript. YS, SO, SI, and SL performed the research. YM and KS analyzed the data. All authors have seen and agreed with the content of the manuscript.

## Conflict of Interest

The authors declare that the research was conducted in the absence of any commercial or financial relationships that could be construed as a potential conflict of interest.

## Publisher’s Note

All claims expressed in this article are solely those of the authors and do not necessarily represent those of their affiliated organizations, or those of the publisher, the editors and the reviewers. Any product that may be evaluated in this article, or claim that may be made by its manufacturer, is not guaranteed or endorsed by the publisher.
