# Supplementary material for: Expression of FLRT2 in Postnatal Central Nervous System Development and After Spinal Cord Injury
Source: Front Mol Neurosci. 2021 Oct 22;14:756264. doi: 10.3389/fnmol.2021.756264 (PMC8569257; doi:10.3389/fnmol.2021.756264)
Supplement: Supplementary file 1 [file Image_1.pdf]

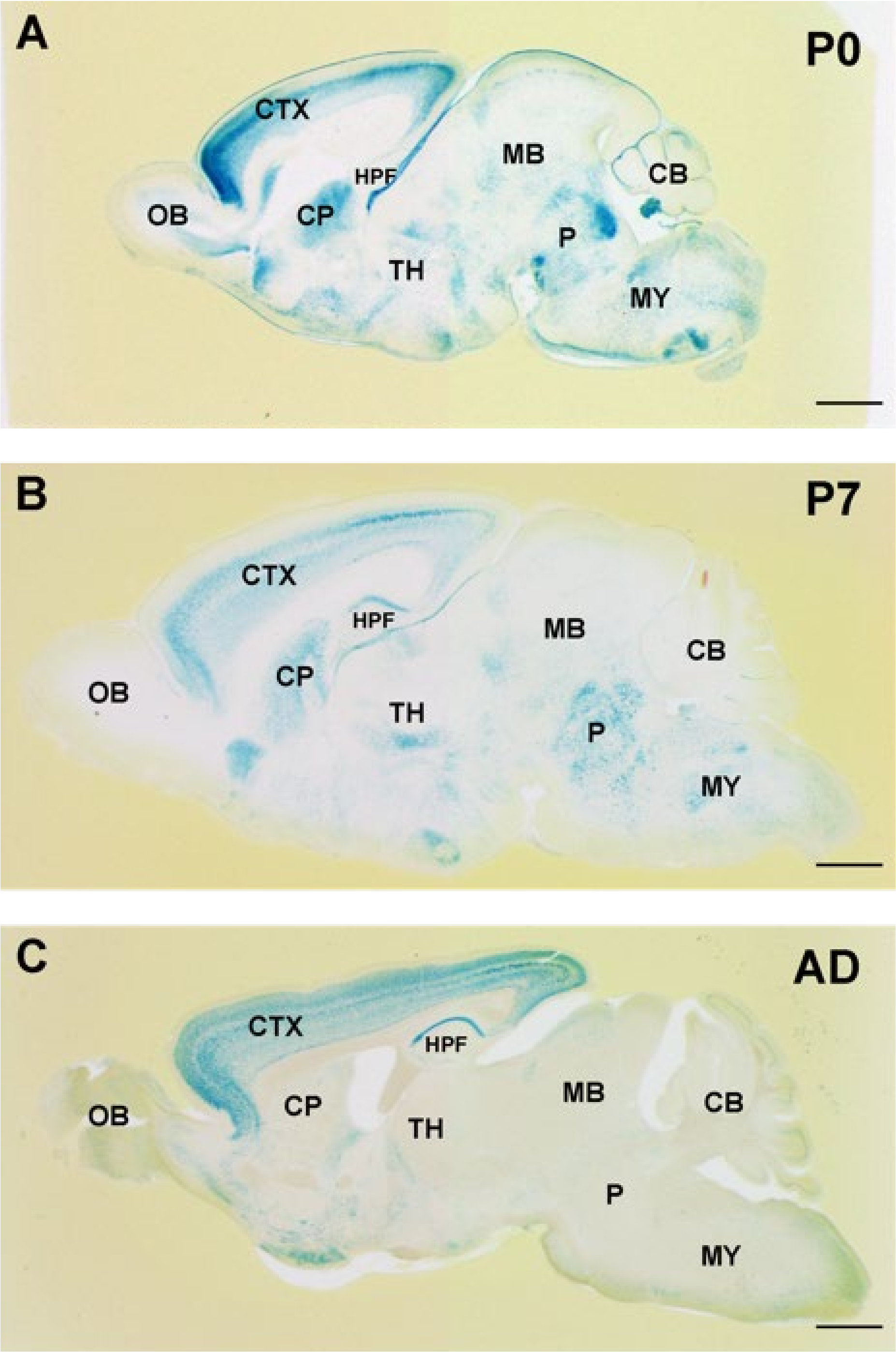

Supplementary Figure 1.

Flrt2-lacZ expression in postnatal stages of brain development, revealed by X-gal staining of sagittal slices at P0 (A), P7 (B), and adulthood (C). Note that FLRT2 expression is strong in prefrontal cortex at P0. CB, cerebellum; CP, caudate putamen; CTX, cerebral cortex; HPF, hippocampal formation; MB, midbrain; MOB, main olfactory bulb; MY, medulla; P, pons; TH, thalamus; VL, lateral ventricle. Scale bars, 1 mm (A-C).

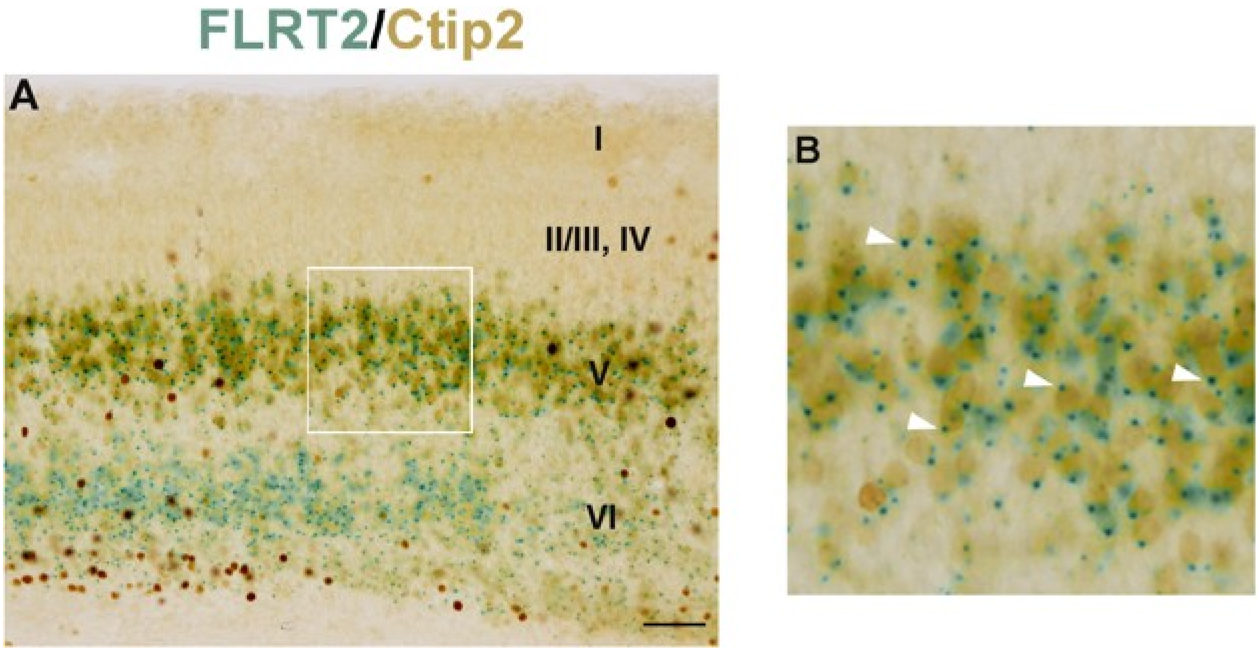

Supplementary Figure 2.

(A, B) Double staining with FLRT2-LacZ and Ctip2 in the cerebral cortex at P0. Note that FLRT2 is expressed in layers V and VI. Scale bar, 50 µm.

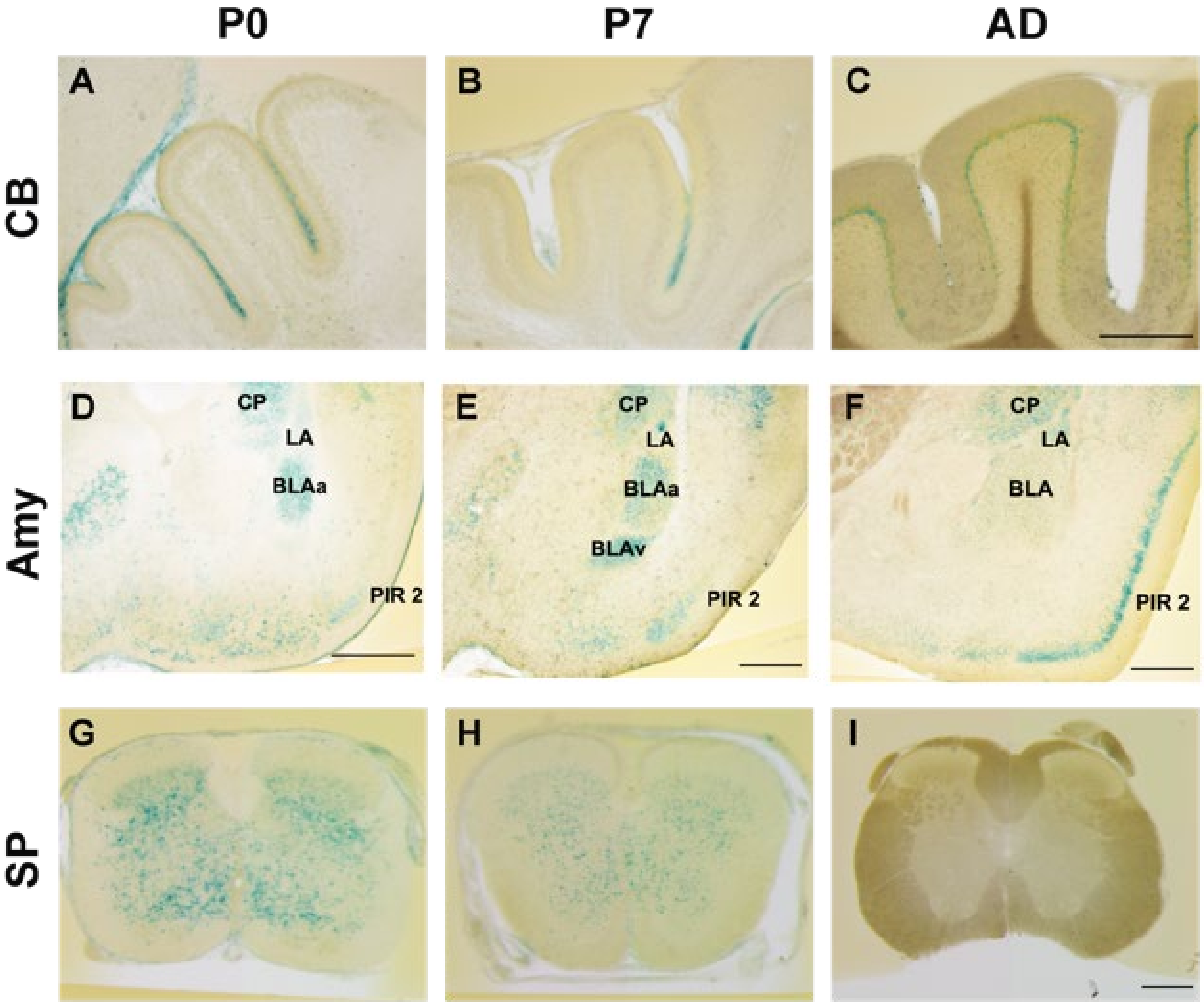

Supplementary Figure 3.

Expression pattern in the cerebellum (CB) (A–C), the amygdala (Amy) (D–F), and the spinal cord (SP) (G-I) at P0, P7, and adulthood, revealed by X-gal staining. Note that FLRT2 is expressed in the Purkinje cell layer in adulthood (C). (G–I) In the spinal cord, FLRT2 expression level was high at P0 and decreased during development. BLAa, basolateral amygdalar nucleus, anterior part; BLAv, basolateral amygdalar nucleus, ventral part; CP, caudate putamen; LA, lateral amygdalar nucleus; PIR 2, piriform area, pyramidal layer. Scale bars, 300 μm (A–I).

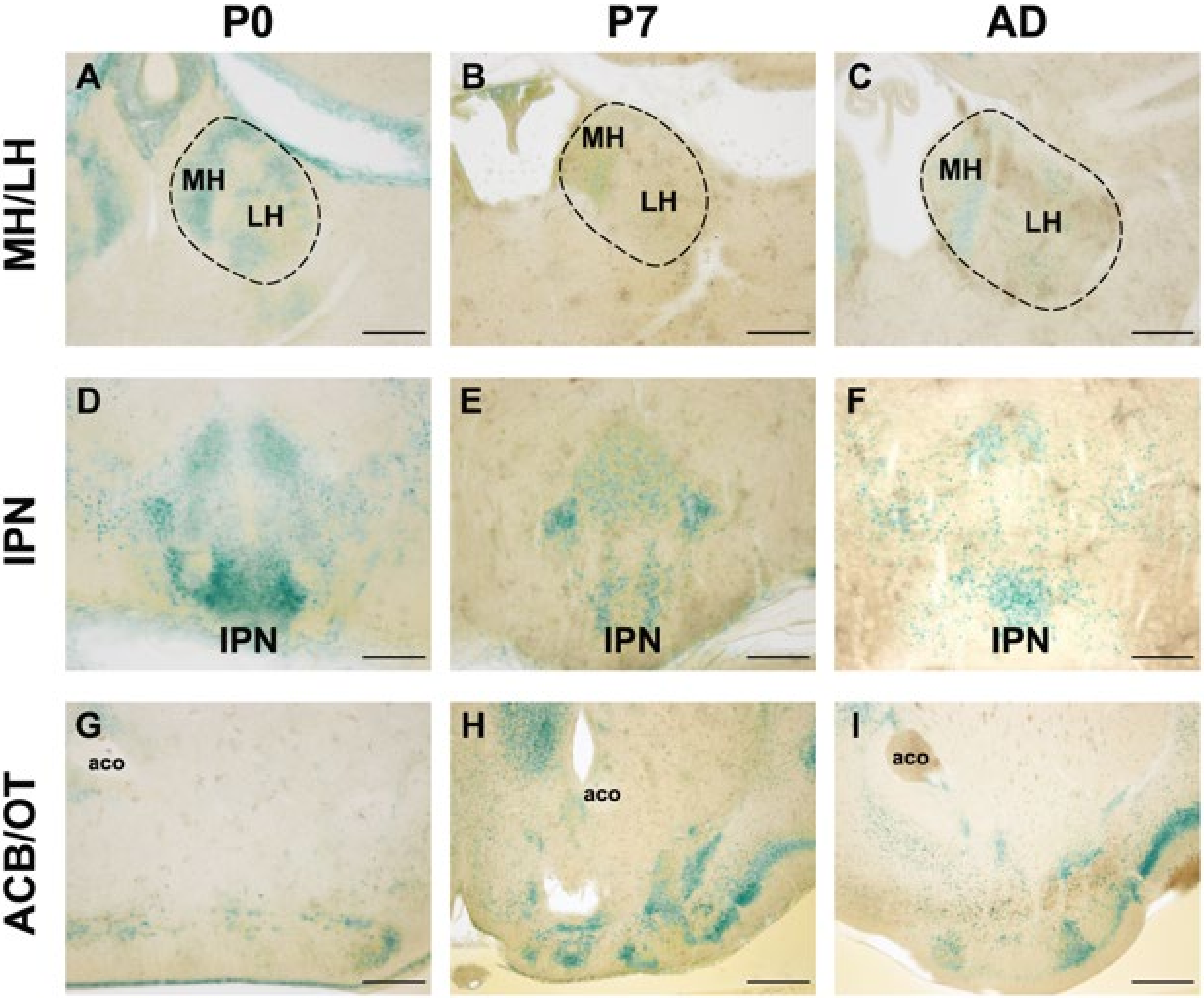

Supplementary Figure 4.

Expression patterns in the medial and lateral habenula (MH and LH) (A-C), interpeduncular nucleus (IPN) (D–F), and olfactory tract (OT) (G–I) at P0, P7, and adulthood, as revealed by X-gal staining. aco, anterior commissure, olfactory limb. Scale bars, 200 µm (A-G); 500 µm (H and I).

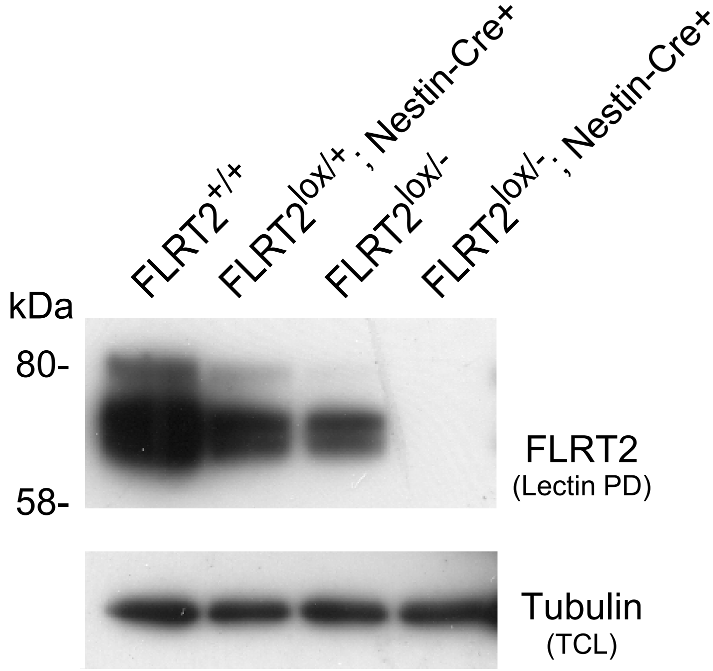

Supplementary Figure 5

Western blot analysis of lectin pull-down (glycoprotein enriched) samples from brain lysates using anti-FLRT2 antibody or corresponding total cell lysates using anti-Tubulin antibody. Please note that FLRT2 protein level decreased half in heterozygotes and could not be detected in conditional knockout mice with null allele.

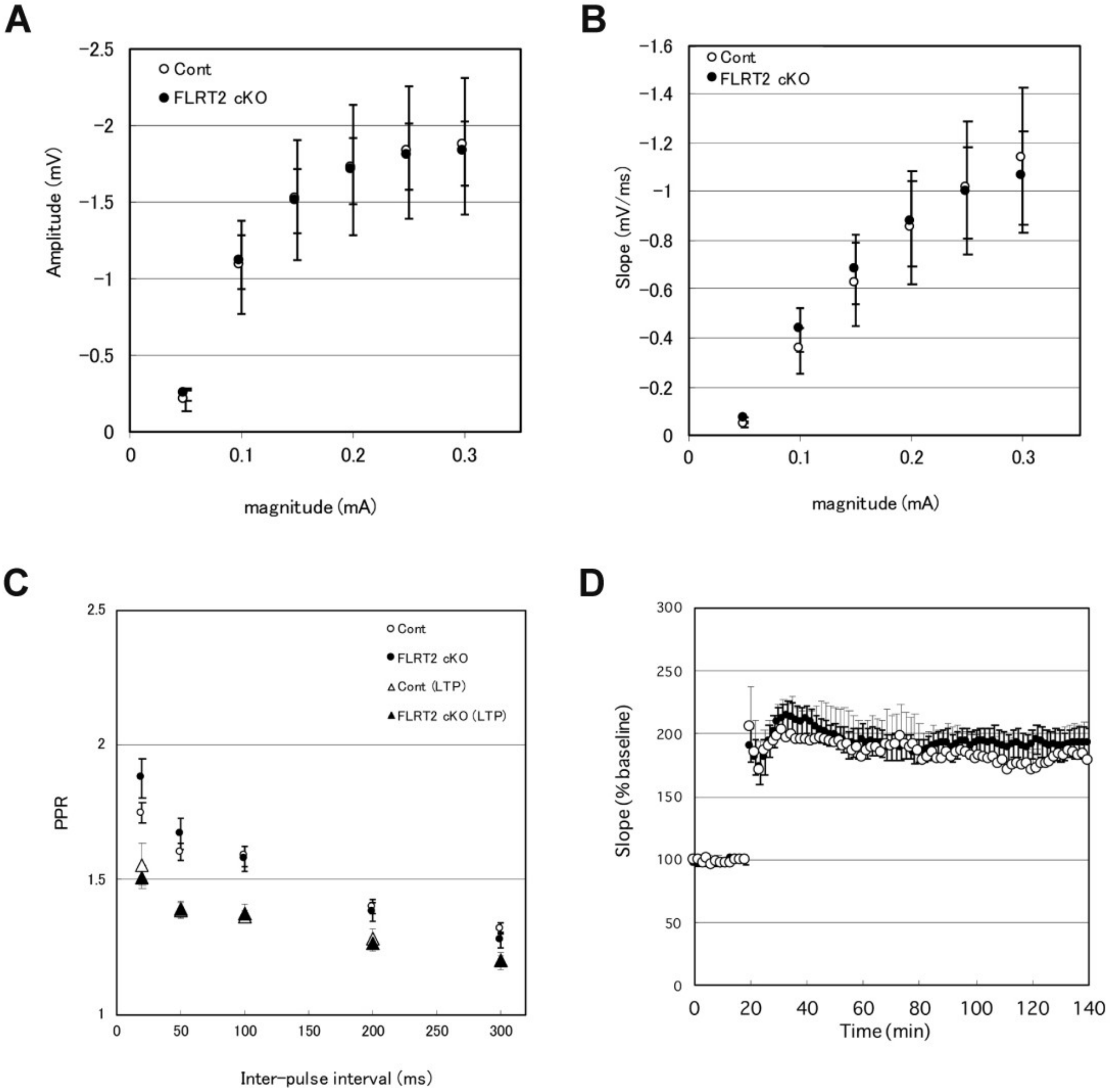

Supplementary Figure 6.

Basal synaptic transmission of CA3-Schaffer collateral-CA1 synapses in hippocampus. (A) fEPSP amplitude. (B) fEPSP slope. n=7 and 12 for control and FLRT2 cKO, respectively. (C) Paired-pulse facilitation before and after LTP induction. n=4 and 11 for control and FLRT2 cKO, respectively. (D) TBS-induced LTP. All parameters were identical between control and FLRT2 cKO. n=4 and 11 for control and FLRT2 cKO, respectively. Error bars represent ± SEM. fEPSP, field excitatory postsynaptic potential; LTP, long-term potentiation; PPR, paired-pulse ratio; TBS, theta-burst stimulation.
